# Evaluating Skellytour for Automated Skeleton Segmentation from Whole-Body CT Images

**DOI:** 10.1148/ryai.240050

**Published:** 2025-02-19

**Authors:** Daniel C. Mann, Michael W. Rutherford, Phillip Farmer, Joshua M. Eichhorn, Fathima Fijula Palot Manzil, Christopher P. Wardell

**Affiliations:** ^1^Department of Biomedical Informatics, University of Arkansas for Medical Sciences, 4301 W Markham St, Little Rock, AR 72205

**Keywords:** CT, Informatics, Skeletal-Axial, Demineralization-Bone, Comparative Studies, Segmentation, Supervised Learning, Convolutional Neural Network (CNN)

## Abstract

**Purpose:**

To construct and evaluate the performance of a machine learning model for bone segmentation using whole-body CT images.

**Materials and Methods:**

In this retrospective study, whole-body CT scans (from June 2010 to January 2018) from 90 patients (mean age, 61 years ± 9 [SD]; 45 male, 45 female) with multiple myeloma were manually segmented using 60 labels and subsegmented into cortical and trabecular bone. Segmentations were verified by board-certified radiology and nuclear medicine physicians. The impacts of isotropy, resolution, multiple labeling schemes, and postprocessing were assessed. Model performance was assessed on internal and external test datasets (362 scans) and benchmarked against the TotalSegmentator segmentation model. Performance was assessed using Dice similarity coefficient (DSC), normalized surface distance (NSD), and manual inspection.

**Results:**

Skellytour achieved consistently high segmentation performance on the internal dataset (DSC: 0.94, NSD: 0.99) and two external datasets (DSC: 0.94, 0.96; NSD: 0.999, 1.0), outperforming TotalSegmentator on the first two datasets. Subsegmentation performance was also high (DSC: 0.95, NSD: 0.995). Skellytour produced finely detailed segmentations, even in low-density bones.

**Conclusion:**

The study demonstrates that Skellytour is an accurate and generalizable bone segmentation and subsegmentation model for CT data; it is available as a Python package via GitHub *(https://github.com/cpwardell/Skellytour)*.

**Keywords:** CT, Informatics, Skeletal-Axial, Demineralization-Bone, Comparative Studies, Segmentation, Supervised Learning, Convolutional Neural Network (CNN)

*Supplemental material is available for this article.*

Published under a CC BY 4.0 license.

See also commentary by Khosravi and Rouzrokh in this issue.

## Graphical abstract

**Figure:**
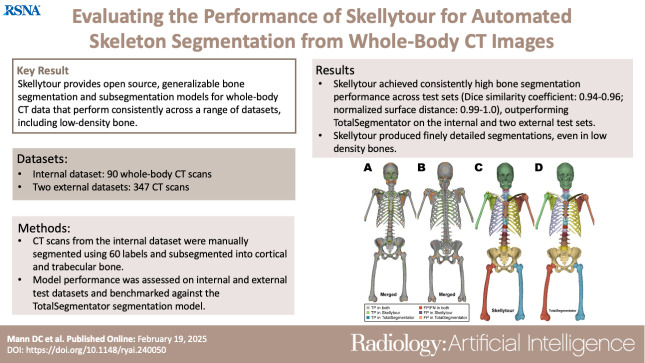

**Summary**

Skellytour provides open-source, generalizable bone segmentation and subsegmentation models for whole-body CT data that perform consistently across a range of datasets, including low-density bone.

**Key Points**

■ Skellytour can segment bone in whole-body CT scans with up to 60 labels, including surgically implanted hardware, and can subsegment bones into cortical and trabecular bone.
■ Skellytour achieved consistently high performance across one internal and two external test sets (Dice similarity coefficient: 0.94, 0.94, 0.96; normalized surface distance: 0.99, 0.999, 1.0, respectively).
■ Skellytour segmentations were quantitatively better with qualitatively higher resolution than a comparable method and can segment low-density bone.

## Introduction

In vivo imaging of bone has been explored since the discovery of radiographs in 1895 (1). CT can focus on specific regions or scan the entire body, resulting in volumetric data at resolutions less than 1 mm^3^. The high radiodensity of bone makes CT data useful for assessing bone mineral density, detecting fractures and osteolytic and osteoblastic lesions, and treatment planning for surgical procedures and radiation therapy. This is particularly important in older patients, who are more likely to be osteoporotic or have diseases affecting the skeleton, such as multiple myeloma.

Segmentation is the process of labeling images at the pixel (two-dimensional, 2D) or voxel (three-dimensional, 3D) level and is crucial in medical imaging analysis, both in clinical and research settings. Manual segmentation is labor-intensive and must be performed by expert radiologists, limiting segmentation to a relatively small user base. Therefore, there is a need to develop automated methods.

For decades, simple heuristic methods have been effective for automating various tasks, such as brain extraction from MRI data (2). Automated bone segmentation in CT images is complex because scanning parameters, field of view, and patient orientation are inconsistent. Additional challenges limiting clinical use of automated bone segmentation methods include low density and potential exclusion of trabecular bone, representation of all bones by a single label, and presence of unwanted features, such as the scanning table and bowel contents, as well as scanning artifacts such as beam hardening and scatter (3).

Prior to machine learning methods, atlas-based segmentation methods were developed using template skeletons. Rigid or affine registration is used to register atlas bones to the target scan, potentially with enforced limits on the distances and articulations between bones. Nonaffine registration allows the atlas to conform to the target skeleton at the cost of preserving bone rigidity. Although Dice similarity coefficient (DSC) values of 0.75 to 0.90 were reported in earlier studies (4,5), no code was made available, and the performance of these methods on external datasets was not tested.

There have been substantial advancements in machine learning methods in the past decade. The key barrier has been gathering enough high-quality labeled data, resulting in either researchers creating their own training data (6,7) or participating in distributed challenges (8–10). The U-Net architecture has proven effective for medical imaging segmentation (11), particularly the nnU-Net Python package (12). For example, TotalSegmentator (13) is an impressively comprehensive general segmentation model that includes bone labels. However, better performance may be achieved by models tailored for specific applications.

We aimed to develop and evaluate the performance of Skellytour, a machine learning model based on nnU-Net for automated bone segmentation in whole-body CT images. Whole-body scans in patients with multiple myeloma were manually segmented using 60 bone labels and subsegmented into cortical and trabecular bone. Skellytour was benchmarked against the TotalSegmentator segmentation model using one internal and two external datasets.

## Materials and Methods

### Datasets

This retrospective study was classified as nonhuman subjects research by the institutional review board of the University of Arkansas for Medical Sciences (UAMS) (under institutional review board approval no. 262472), and formal review was waived. Three retrospective datasets were used to develop and test the proposed model: a local dataset to train and test models and two external datasets to further assess performance.

**Skellytour dataset.**

The internal dataset comprised 90 whole-body CT scans from patients with multiple myeloma that were collected from June 2010 to January 2018 as part of routine PET/CT imaging at UAMS with two machines at three resolutions. This collection was intentionally heterogeneous and intended to broadly represent patients with multiple myeloma and their imaging data to train generalizable models. It is estimated that 90% of the general population has seven cervical, 12 thoracic, and five lumbar vertebrae (14,15); thus, patients with atypical vertebral counts were excluded.

**Large-Scale Vertebrae Segmentation Challenge dataset.**

The first external dataset was the Large-Scale Vertebrae Segmentation Challenge (VerSe) dataset, generated between January 2013 and March 2020 to train vertebrae segmentation models in CT scans (8,9,16). It contains 374 scans and manual segmentations for 355 patients from multiple sites and scanners at multiple resolutions. Most images show a subset of vertebrae, with only nine scans showing a complete spine from C1 to L5. We excluded 63 scans with atypical vertebral counts, leaving a final dataset of 311 scans.

**TotalSegmentator dataset.**

The TotalSegmentator dataset was the second external dataset used for model testing. Generated to develop the TotalSegmentator model (13), this dataset contains 1228 CT scans representing 36 different study types from eight sites with 16 scanners, all resampled to 1.5-mm^3^ isotropic voxels. Segmentations are the manually refined results of an iterative annotation workflow involving several machine learning models. We manually reviewed the test set segmentations and excluded 29 of them for bone segmentation defects, leaving 36 segmentations. Common defects included surgically implanted hardware labeled as bone and label mismatch in the vertebrae (Fig S1). Further details of exclusion criteria are given in Table S2.

### Annotation Strategy and Postprocessing

**Figure 1:**
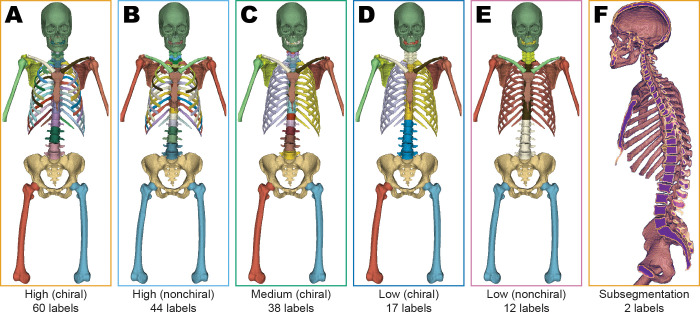
**(A–E)** Reference standard manually annotated segmentations from the Skellytour training data labeled with each of the five segmentation labeling schemes used in developing Skellytour. **(F)** Sagittal view of the subsegmentation scheme, which divides bones into cortical and trabecular regions.

3D Slicer (17) was used to create 60 binary masks per patient, comprising 59 individual bones and one label for high radiodensity features, including surgically implanted hardware, metal clothing elements, and dentures. Additionally, bones were subsegmented into cortical and trabecular regions. Segmentations were verified by a board-certified radiologist (J.M.E., 4 years of experience) and a board-certified nuclear medicine physician (F.F.P.M., 9 years of experience). Binary masks were compiled into five labeling schemes with increasing numbers of labels (Fig 1; Tables 1, S3), which were the reference standards for assessing model performance. We developed three postprocessing Python scripts. The first was used to infer chirality for pairs of bones (eg, femurs, humeri) in nonchiral labeling schemes, assigning each bone as left or right based on the relative position of their centroids. The second retains the largest island of connected voxels and removes small, noncontiguous islands, which are likely false-positive findings. The third ensures that predicted cortical and trabecular subsegmentations match bone segmentations and that the exterior surface is composed of cortical bone.

**Table 1:**
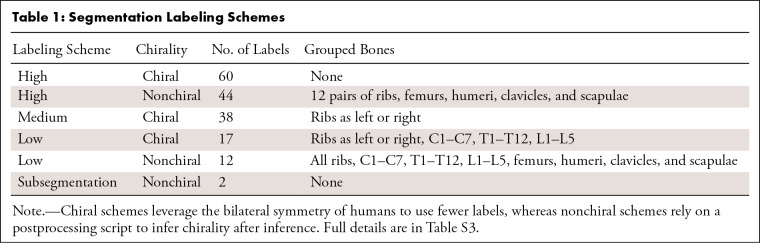
Segmentation Labeling Schemes

### Models, Data Splits, Training, and Testing

We tested four model architectures in nnU-Net: 2D, 3D low resolution, 3D high resolution, and a cascade that runs the low- and high-resolution 3D models sequentially. Training data were split into six groups of 15 scans, each balanced for patient and imaging characteristics. Models were trained for 1000 epochs using fivefold cross-validation, with 60 training and 15 validation scans per fold. The ensemble of all folds was evaluated for each model using the same test set of 15 scans, which was completely independent and not used when training or tuning models. Data augmentation using mirroring was disabled when training and testing models using chiral labeling schemes. The cortical and trabecular bone subsegmentation model was trained using 25 scans with a test set of five scans using fivefold cross-validation. It runs directly on the input images, and a postprocessing script ensures that it conforms exactly to the predicted bone segmentations and that the exterior surface is composed of cortical bone. Subsegmentation is a simpler task with only two labels, allowing training and testing with fewer scans than the main model. Model and labeling scheme selection used models trained with nnU-Net version 1.7.1, and the model compared with TotalSegmentator used nnU-Net version 2.3.1. To investigate the impact of isotropic voxels on model performance, we used the SimpleITK Python package to resample the Skellytour dataset to isotropic voxels using cubic B-spline and nearest neighbor methods for images and segmentations, respectively, and trained a model to predict both isotropic and nonisotropic inputs. This was compared against a model trained using the original nonisotropic data, all using the low, nonchiral labeling scheme with 12 labels. To assess whether data augmentation via mirroring affected segmentation quality, we compared predicted segmentations from models using the equivalent chiral and nonchiral versions of the high and low labeling schemes. Models were trained for each scheme, and nonchiral segmentations were converted to chiral schemes with a postprocessing script.

### Metrics and Statistical Analysis

Predicted and reference standard segmentations were compared using the DSC as an overlap metric and three distance-based metrics, as follows: *(a)* Normalized surface distance (NSD) gives the proportion of voxels within a specified distance of the reference standard. A 3-mm tolerance was used because it is the lowest common multiple of the resolutions of our data, and all TotalSegmentator data have 1.5-mm voxels. *(b)* Average symmetric surface distance gives the average distance in millimeters between the perimeters of reference standard and predicted segmentations. *(c)* The 95th percentile of the Hausdorff distance was defined as the 95th percentile of the distances between reference standard and predicted segmentations. Metrics were calculated using Python scripts, and analysis was performed using R Statistical Software (version 4.3.2; R Core Team). Aggregate metrics are reported as the median of all labels, with a 95% CI using the two-sided exact method in the MedianCI function of the DescTools R package, and were compared using Wilcoxon rank sum tests. A *P *value less than .05 was considered statistically significant.

### Code Availability

Skellytour code and models are available via GitHub *(https://github.com/cpwardell/Skellytour)*.

## Results

### Dataset Characteristics

The internal Skellytour dataset patient and imaging characteristics are in Table 2, with expanded data in Table S1. There were 90 patients, with 45 male and 45 female patients, and a mean age of 61 years ± 9 (SD). The external VerSe dataset contained 311 scans from 294 patients, with 142 male and 152 female patients, and a mean age of 59 years ± 17. The external TotalSegmentator dataset contained 36 patients, with 25 male and 11 female patients and a mean age of 63 years ± 12.

**Table 2:**
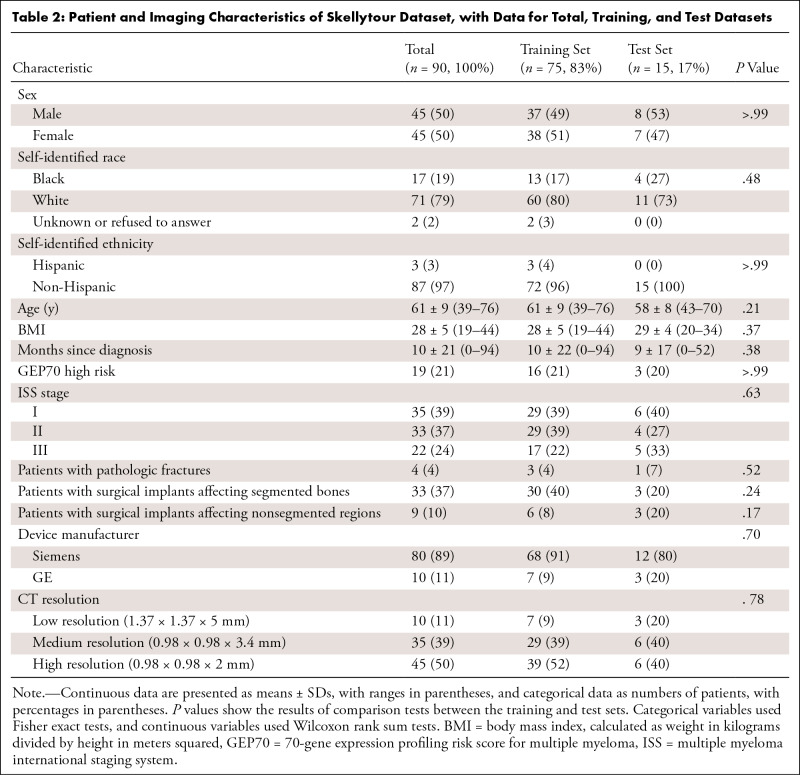
Patient and Imaging Characteristics of Skellytour Dataset, with Data for Total, Training, and Test Datasets

### Comparison of nnU-Net Models

To select the optimum model, we trained 2D, 3D low-resolution, 3D full-resolution, and 3D cascade models using the low, nonchiral labeling scheme with 12 labels. There was no evidence of performance differences between models trained with nnU-Net version 1.7.1 or 2.3.1 (Fig S2). The full-resolution 3D model produced the best metrics across all labels (Fig S3). Manual inspection revealed that 2D models frequently produced false-positive results in single sections that 3D models did not. Therefore, we opted to train only 3D full-resolution models.

### Impact of Isotropic Data and Resolution

**Figure 2:**
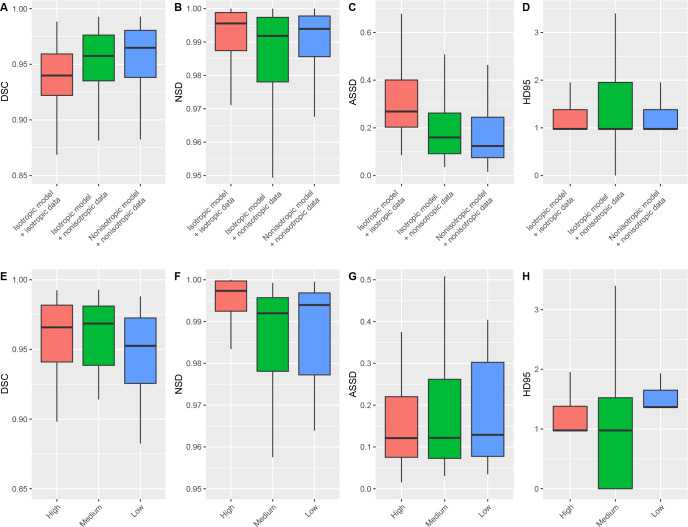
Effects of isotropy (top row) and resolution (bottom row) on model performance. **(A–D)** DSC, NSD, ASSD, and HD95 values show that converting data to isotropic voxels lowers model performance compared with a model trained with the native nonisotropic data. **(E–H)** DSC, NSD, ASSD, and HD95 values show minor differences with different input resolutions. Models generally achieved peak performance with higher-resolution data. Box plots show the median (horizontal line) and the IQR (box edges). Whiskers extend to the value at most 1.5 times the IQR. ASSD = average symmetric surface distance, DSC = Dice similarity coefficient, HD95 = 95th percentile of the Hausdorff distance, NSD = normalized surface distance.

We found that isotropic data had detrimental effects, as the model trained on isotropic data had lower performance on both isotropic (DSC: 0.94 [95% CI: 0.93, 0.95]) and nonisotropic data (DSC: 0.96 [95% CI: 0.95, 0.96]) compared with the nonisotropic model with nonisotropic data (DSC: 0.96 [95% CI: 0.96, 0.97], *P* < .001) (Fig 2A–2D). Additionally, we found models had slightly improved performance with higher-resolution data (high DSC: 0.97 [95% CI: 0.96, 0.97], medium DSC: 0.97 [95% CI: 0.95, 0.98], low DSC: 0.95 [95% CI: 0.94, 0.97]; *P* = .04) (Fig 2E–2H). We therefore opted to keep the nonisotropic native resolutions of our data.

### Comparison of Chiral and Nonchiral Labeling Schemes

We found no significant difference in metrics between chiral and nonchiral models. Also, patient orientation could interfere with postprocessing, so we opted to use only chiral labeling schemes. We also found that models with fewer labels produced better results (Fig S4).

### Comparison of Chiral Labeling Schemes

**Figure 3:**
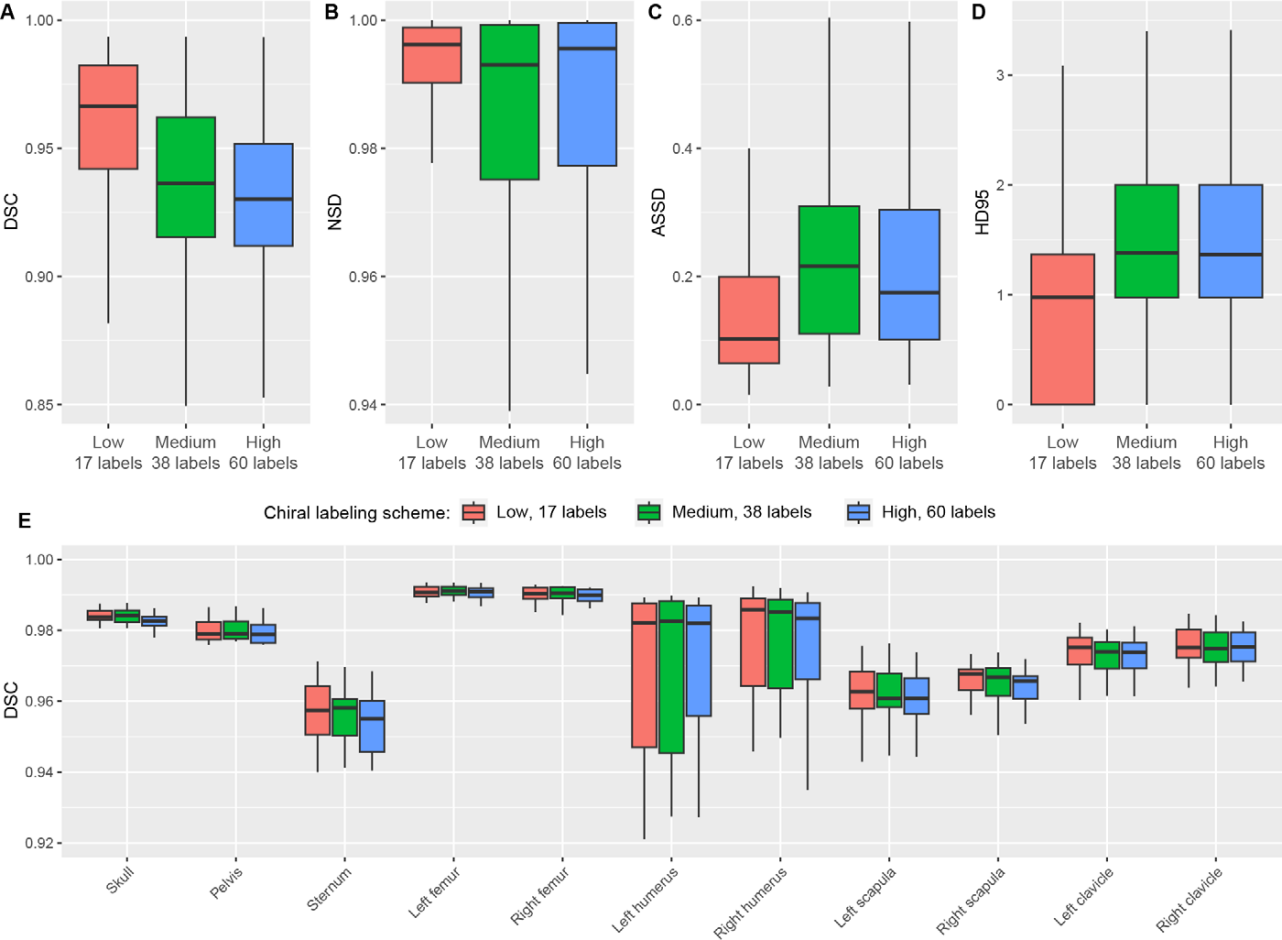
Performance metrics for labeling schemes. **(A–D)** Models with fewer labels have higher DSC and NSD and lower ASSD and HD95. **(E)** DSC for individual bones is similar between models, with lower scores for smaller and more complex bones. Box plots show the median (horizontal line) and the IQR (box edges). Whiskers extend to the value at most 1.5 times the IQR. ASSD = average symmetric surface distance, DSC = Dice similarity coefficient, HD95 = 95th percentile of the Hausdorff distance, NSD = normalized surface distance.

We trained models using three chiral labeling schemes: high (60 labels), medium (38 labels), and low (17 labels), hypothesizing that fewer labels may lead to improved segmentations. We found a small decrease in metrics as the number of labels increased (Fig 3A–3D). This pattern held when considering only the 11 labels shared among all three labeling schemes (Fig 3E) and the 24 vertebrae in the high and medium schemes, suggesting that having more labels detrimentally affects performance across all labels. The ribs and vertebrae are relatively small and similar bones that are packed closely together, leading to label mismatch, where some bones are erroneously labeled with a neighboring label or mixture of labels. This occurred most frequently in the transverse and spinous processes of thoracic vertebrae and affected three of 15 (20%) segmentations in the high scheme and four of 15 (27%) segmentations in the medium scheme. The same issue was found in the ribs using the high scheme in eight of 15 (53%) segmentations. We therefore opted for the medium labeling scheme as a default, as it balances comprehensive and high-quality segmentation.

### Postprocessing Bone Segmentations

Only the largest island for all labels except the ribs is retained; all small, noncontiguous regions are removed. For ribs, the first 12 islands of at least 1000 mm^3^ are retained. This threshold was determined empirically using the rib volumes in the Skellytour dataset. No further postprocessing was applied to the artifacts label. Postprocessing led to no evidence of differences in metrics compared with segmentations without postprocessing using the Skellytour test set (Fig S5).

### Subsegmentation of Bone into Cortical and Trabecular Regions

Non-postprocessed subsegmentation achieved high DSC (0.92 [95% CI: 0.82, 0.94]) and NSD values (0.99 [95% CI: 0.97, 0.99]). Postprocessing significantly improved DSC (0.95 [95% CI: 0.94, 0.98], *P* < .001) but not NSD (0.995 [95% CI: 0.98, 0.999], *P* = .83). Metrics are shown in Figure S6, and DSC values for each bone are in Table S4.

### Runtime and Resource Utilization

Runtime and maximum RAM and GPU RAM usage for Skellytour and TotalSegmentator were calculated for three example images (Table 3). Runtime and resource utilization increased for all methods with the size of the input volume and number of labels.

**Table 3:**
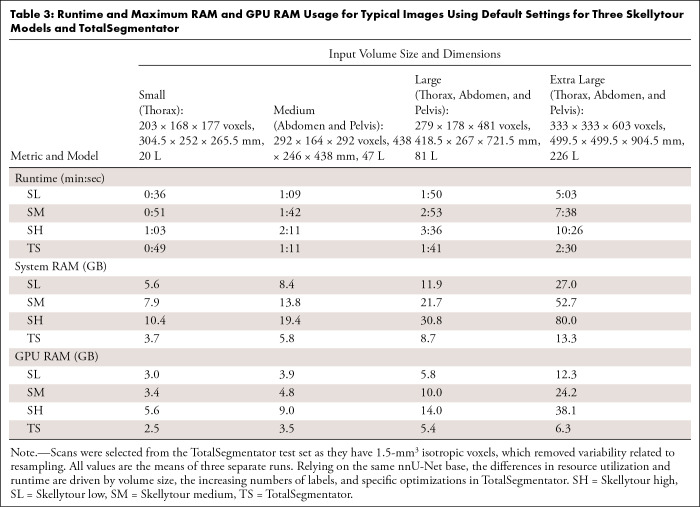
Runtime and Maximum RAM and GPU RAM Usage for Typical Images Using Default Settings for Three Skellytour Models and TotalSegmentator

### Comparison of Skellytour and TotalSegmentator Performance on Internal and External Test Sets

We benchmarked results using our final 3D high-resolution model using the medium scheme with postprocessing against results from the default 1.5-mm^3^ model in TotalSegmentator (version 2.1.0) using three datasets: the Skellytour internal test set (*n* = 15) and the VerSe (*n* = 311) and TotalSegmentator (*n* = 36) external test sets. We only considered labels in each reference standard dataset.

**Skellytour test set.**

**Figure 4:**
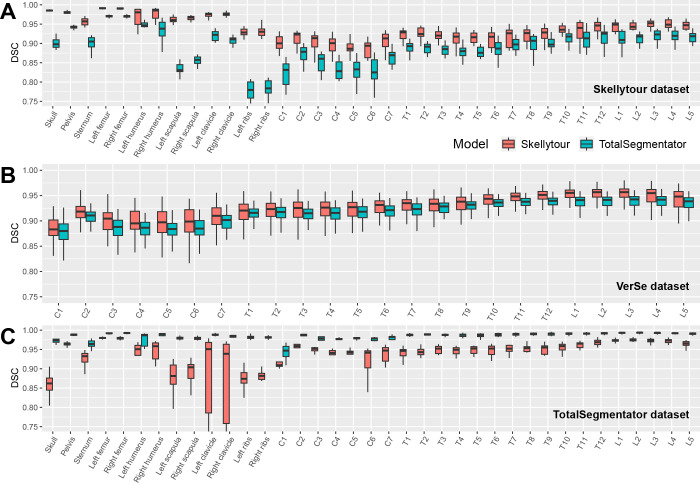
DSC comparisons between Skellytour and TotalSegmentator for individual bones. **(A, B)** In the Skellytour and VerSe test sets, Skellytour consistently outperformed TotalSegmentator. **(C)** In the TotalSegmentator test set, Skellytour was outperformed by TotalSegmentator. For equivalent normalized surface distance, average symmetric surface distance, and 95th percentile of the Hausdorff distance plots, see Figures S7–S9. Box plots show the median (horizontal line) and the IQR (box edges). Whiskers extend to the value at most 1.5 times the IQR. DSC = Dice similarity coefficient, VerSe = Large-Scale Vertebrae Segmentation Challenge.

**Figure 5:**
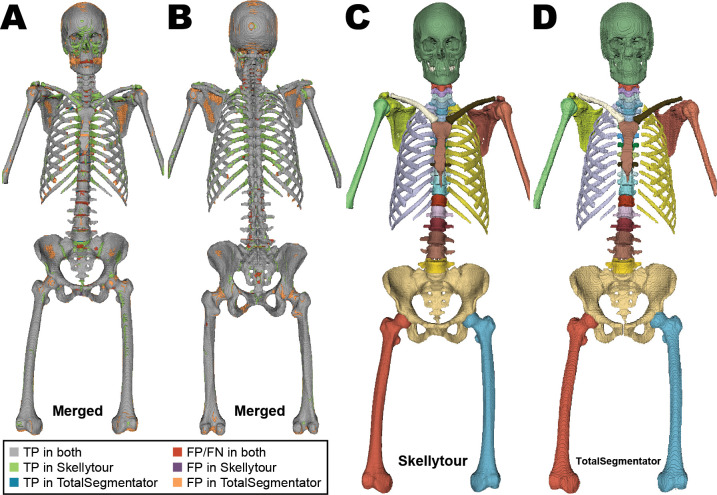
**(A, B)** Anterior and posterior views of bones segmented by Skellytour and TotalSegmentator. Colors represent differentially segmented regions with a volume of at least 50 mm^3^. Recurrent differences include the proximal and distal ends of ribs and the costal and distal surfaces of the scapulae (Figs S10, S11). **(C, D)** Anterior views of predicted segmentations by **(C)** Skellytour and **(D)** TotalSegmentator illustrate the effect of low-density regions, particularly superior regions of the scapulae and left acromion, and the finer detail visible in Skellytour. Comparisons of segmentations in pathologic regions are in Figures S12 and S13. FN = false negative, FP = false positive, TP = true positive.

Skellytour achieved high DSC and NSD metrics (DSC: 0.94 [95% CI: 0.93, 0.94], NSD: 0.99 [95% CI: 0.99, 0.995]), significantly outperforming TotalSegmentator (DSC: 0.90 [95% CI: 0.89, 0.90], NSD: 0.97 [95% CI: 0.96, 0.97]) (*P* < .001 for both) (Fig 4A). Manual inspection revealed several regions that only Skellytour accurately segmented. These included the heads of ribs that articulate to the vertebrae and portions of the superior border, acromion, and coracoid process of the scapulae (Figs 5, S10, S11). TotalSegmentator also produced recurrent artifacts, segmenting nonbone regions of the scapulae (Fig S11). Both models suffered from label mismatch in the vertebrae at similar rates (Skellytour: four of 15 [27%], TotalSegmentator: three of 15 [20%]). Additionally, Skellytour successfully labeled bone cement in vertebrae, whereas TotalSegmentator left this unlabeled, resulting in voids within the vertebral body. There were also differences related to pathology, with Skellytour more accurately segmenting bones with osteolytic lesions and expansile rib lesions (Figs S12, S13). Although both models output segmentations at the same resolution as the input image, Skellytour results qualitatively appeared to have a higher resolution (Fig S14).

**VerSe test set.**

The 311 VerSe segmentations could be used to test performance only in vertebrae. Skellytour metrics (DSC: 0.94 [95% CI: 0.94, 0.94], NSD: 0.999 [95% CI: 0.999, 0.999]) were higher than those of TotalSegmentator (DSC: 0.93 [95% CI: 0.93, 0.93], NSD: 1.0 [95% CI: 1.0, 1.0]), (DSC: *P* < .001) (Fig 4B). Label mismatch was assessed by manually inspecting a subset of 50 segmentations and occurred at the same rate in seven of 50 (14%) of cases for both models.

**TotalSegmentator test set.**

Initially, both models produced many segmentations with very low DSC. Manual review revealed that bones appearing at the edges of volumes were often incorrectly labeled or not labeled at all in either the reference standard or predicted segmentations. To ameliorate this, we calculated the mean volume of each complete bone using the Skellytour dataset and excluded segmentations less than 20% of these volumes. This removed 13% of the total segmentations and had a non–statistically significant effect on the metrics. Skellytour DSC and NSD metrics (DSC: 0.96 [95% CI: 0.96, 0.96], NSD: 1.0 [95% CI: 0.999, 1.0]) were lower than TotalSegmentator metrics (DSC: 0.99 [95% CI: 0.99, 0.99], NSD: 1.0 [95% CI: 1.0, 1.0]) (*P* < .001) (Fig 4C). The label mismatch rate in vertebrae was low, with two cases (6%) for each model.

## Discussion

Skellytour is a general-purpose bone segmentation tool for CT data, specifically focused on patients with low bone density and surgically implanted hardware, that can also subsegment bones into cortical and trabecular regions. The internal training and test data of 90 manually segmented skeletons used a heterogeneous set of older patients with multiple myeloma and was balanced for both patient characteristics, including sex and self-identified race, and imaging characteristics, including resolution and manufacturer. We also tested our model using two external datasets and compared results against an alternative model, TotalSegmentator. Skellytour and TotalSegmentator are alike as they are nnU-Net based and produced high scores across all three test sets. However, Skellytour was more consistent, not only scoring higher on its own test set (DSC: 0.94 vs 0.90, *P* < .001) but also in the large VerSe dataset (DSC: 0.94 vs 0.93, *P* < .001). This is notable because VerSe was also manually annotated and shares no overlap with other datasets. The benchmarks demonstrate that Skellytour is generalizable as it produced consistent metrics across all three test sets (DSC: 0.94, 0.94, 0.96 vs 0.90, 0.93, 0.99), regardless of input resolution or field of view.

The performance disparities between Skellytour and TotalSegmentator possibly stem from differences in training and inference methods, and either model may be overfitted to its respective training data. The Skellytour data consist only of whole-body scans that were manually annotated and verified by human experts. TotalSegmentator was trained using data with varying fields of view, using an iterative annotation workflow; small numbers of scans were manually annotated or predicted using public models and manually refined, with the number of scans increasing at each iteration. This may explain the recurrent artifacts we found by manual review, which resulted in excluding 29 of 65 (45%) of the TotalSegmentator test data. During inference, Skellytour uses an ensemble of five models that include all labels, leading to higher resource utilization and slightly longer runtimes, whereas TotalSegmentator runs single folds of five models with fewer labels and combines them. Although both algorithms output segmentations at the same resolution as the input image, Skellytour segmentations qualitatively appear to be smoother and have a higher resolution, likely because TotalSegmentator resamples all inputs to the same 1.5-mm^3^ isotropic resolution for inference.

Overlap and surface metrics such as DSC and NSD are summary statistics and do not capture all the information. Closer inspection of segmentations revealed that differences in metrics were often driven by differences among the training data for each model. The intentional choice of using patients with multiple myeloma with osteolytic lesions and low bone density allowed Skellytour to accurately segment bones and regions that TotalSegmentator could not. Examples in the Skellytour data include the scapulae, osteolytic lesions in the skull, and expansile lesions in the ribs. We found regions of soft tissue were recurrently included in segmentations of the scapulae by TotalSegmentator. It is also notable that surgically implanted hardware was either unlabeled or labeled by TotalSegmentator as the bones that they replace or augment, although TotalSegmentator does provide a separate model to segment hip implants.

This study had important limitations. First, label mismatch was a common issue in both models and was found at similar rates between each one. However, it occurred at different rates in each test set, most frequently in the Skellytour data. This may be because Skellytour images contained a full set of vertebrae, whereas others contained subsets and fewer opportunities for error. Label mismatch is likely a fundamental issue with nnU-Net among clusters of highly similar bones. Our immediate solution is to use labeling schemes that group bones, and in the long term, we will train new models as technology improves and include these in future releases. For example, transformer-based extensions to U-Net show substantial promise as their context-sensitive nature may ameliorate label mismatch (18,19). Second, high resource utilization is an issue, as during inference, Skellytour uses an ensemble of five models that include all labels, leading to higher resource utilization and slightly longer runtimes, whereas TotalSegmentator runs single folds of five models with fewer labels and combines them.

In conclusion, this study demonstrates the value of high-quality hand-annotated datasets in producing generalizable, task-specific segmentation models. Skellytour achieved high, consistent bone segmentation performance on internal and external datasets. It provides several labeling schemes, labels surgically implanted hardware, subsegments bones into cortical and trabecular regions, and segments bones with low density and osteolytic lesions. Future work will focus on increasing the size of the training data and the number of labels while decreasing resource utilization.
